# Ressonância Magnética Cardíaca de Estresse em Idosos: Fornece as Respostas?

**DOI:** 10.36660/abc.20220385

**Published:** 2022-07-07

**Authors:** Vera Vaz Ferreira, Boban Thomas

**Affiliations:** 1 Centro Hospitalar Universitário de Lisboa Central Hospital de Santa Marta Serviço de Cardiologia Lisboa Portugal Serviço de Cardiologia, Hospital de Santa Marta, Centro Hospitalar Universitário de Lisboa Central, Lisboa – Portugal; 2 Hospital Cruz Vermelha Portuguesa Heart Center Lisboa Portugal Heart Center, Hospital Cruz Vermelha Portuguesa, Lisboa – Portugal

**Keywords:** Espectroscopia de Ressonância Magnética/métodos, Doença Arterial Coronariana/cirurgia, Idoso, Diagnóstico por Imagem/tendências, Teste de Esforço/métodos

***‘A vida é a arte de tirar conclusões suficientes de premissas insuficientes’***

**Samuel Butler**

Após o ensaio clínico ISCHEMIA, apesar do debate em curso, os médicos precisam contemplar todas as estratégias para pacientes com doença cardíaca isquêmica crônica estável (DCICE).^1^ O manejo da DCICE tem dois objetivos – melhorar o prognóstico e/ou aliviar os sintomas. A maioria dos estudos demonstrou que a revascularização oferece maior alívio de sintomas comparativamente com a terapêutica médica optimizada isoladamente, mas dados sobre os hard outcomes ainda são escassos. Portanto, é importante melhorar nossas ferramentas de tomada de decisão clínica na seleção de pacientes para revascularização. Isso se torna particularmente importante quando selecionamos pacientes idosos para revascularização, porque a relação risco-benefício é provavelmente mais tênue do que em uma coorte mais jovem. Os idosos apresentam dificuldades na avaliação da DCICE por múltiplos motivos. Um refinamento é a seleção adequada do método de avaliação da DCICE. Existe uma panóplia de técnicas não invasivas de imagem, incluindo tomografia computadorizada cardíaca, ecocardiografia, técnicas de medicina nuclear e ressonância magnética cardíaca (RMC). Combinar o paciente certo e o método certo de avaliação seguido da estratégia terapêutica certa melhora os resultados. Na medicina clínica, o importante não é o quanto fazemos, mas o quão bem nossos pacientes se saem depois do que fazemos.

A RMC de perfusão de estresse é um recurso valioso com alto valor preditivo negativo naqueles que não apresentam defeitos de perfusão, independentemente da presença ou ausência de doença arterial coronariana.^2^ Existem poucos estudos sobre o valor prognóstico da RMC de perfusão de estresse com adenosina em pacientes idosos com ou sem DCICE estabelecida. O estudo de Boonyasirinant e Kaolawanich,^3^ embora não seja o primeiro, é uma adição bem-vinda ao repositório de literatura existente que fornece uma razão convincente para o uso de RMC de estresse para a avaliação de isquemia agora abordando a faixa etária idosa.^3^ Seus achados demonstram que, embora os dados clínicos tenham sido combinados com informações sobre a função ventricular esquerda, nenhum valor incremental foi observado em comparação com os dados clínicos isolados na previsão de eventos cardíacos graves. A presença de isquemia detectada pela RMC ajudou a prever eventos de modo significativamente melhor. O poder preditivo não foi aumentado com a adição de informação do realce tardio com gadolínio. Esteban-Fernandez et al. verificaram que pacientes idosos com grau moderado ou grave de isquemia na RMC de estresse têm maior risco de ter um evento durante o seguimento.^4^ Juntos, esses dois estudos fornecem evidências para o uso da RMC de estresse com adenosina na previsão de resultados em pacientes idosos. Uma característica notável de ambos os estudos é a ausência de eventos adversos significativos de adenosina. No entanto, o ensaio clínico ISCHEMIA refletiu a disponibilidade limitada de equipamentos e experiência, onde a RMC foi o método menos escolhido para avaliar a isquemia (5%), refletindo a prática do mundo real.

Como quase todos os estudos significativos em medicina cardiovascular, a escolha dos desfechos é discutível. Este estudo definiu eventos cardíacos graves como infarto do miocárdio não fatal e mortalidade cardíaca. Com a terapia atual, a maioria dos infartos do miocárdio não é fatal, e as definições em evolução de infarto do miocárdio o tornam um alvo móvel. Em segundo lugar, embora todos tentemos usar a medicina baseada em evidências como base de nossa prática clínica, precisamos lembrar que a medicina é essencialmente um negócio de varejo, como observado eloquentemente por Atul Gawande.^5^ Como tratamos cada paciente individualmente e não a população em si, a significância estatística nem sempre é sinônimo de relevância clínica para o paciente individual.

Outro ponto importante a ser lembrado é que o risco de eventos cardíacos adversos, inerente à presença de marcadores como isquemia, pode ser amenizado com TMI adequada. Nenhuma informação clara sobre a terapia usada nos dois grupos é fornecida. Notou-se uma diferença muito significativa nas taxas de tabagismo entre aqueles com e sem isquemia. Embora não seja estatisticamente significativo para ser inserido no modelo, os médicos sabem como o tabagismo continuado contribui para os eventos clínicos. Na prática clínica do mundo real, a imagem geralmente é solicitada quando a revascularização é contemplada naqueles com DCICE conhecida e não necessariamente para estratificar o risco dos pacientes. Nenhuma técnica de imagem para avaliar DCICE é o santo graal, e todas são essencialmente complementares. Com o envelhecimento da população mundial, devemos buscar as melhores formas de avaliar e gerenciar a DCICE.

O Dr. Thomas atuou como Consultor do Centro de Coordenação Clínica do estudo ISCHEMIA no NYU Langone Medical Center.
