# Supplementary material for: Ressonância Magnética Cardíaca de Estresse em Idosos: Fornece as Respostas?
Source: Arq Bras Cardiol. 2022 Jul 7;119(1):107–8. [Article in Portuguese] doi: 10.36660/abc.20220385 (PMC9352139; doi:10.36660/abc.20220385)
Supplement: Translation

# Translation: Stress CMR in the Elderly: Does It Provide the Answers?

**Authors:** Vera Vaz Ferreira, Boban Thomas

**Affiliations:** 1 Lisboa Portugal Serviço de Cardiologia, Hospital de Santa Marta, Centro Hospitalar Universitário de Lisboa Central, Lisboa – Portugal; 2 Lisboa Portugal Heart Center, Hospital Cruz Vermelha Portuguesa, Lisboa – Portugal

**Keywords:** Magnetic Resonance Spectroscopy/methods, Coronary Artery Disease/surgery, Aged, Diagnostic,Imaging, Exercise Test

***‘Life is the art of drawing sufficient conclusions from insufficient premises’***

**Samuel Butler**

After the ISCHEMIA clinical trial, despite the ongoing debate, clinicians need to contemplate all strategies for patients with chronic stable ischemic heart disease (SIHD).^1^ The management of SIHD has two aims – improve prognosis and/or relieve symptoms. Most studies have shown that revascularisation provides greater symptom benefits than optimal medical therapy (OMT) alone, but data on hard outcomes have been elusive. Therefore, improving our clinical decision-making tools in selecting patients for revascularization is important. This becomes particularly important when we select elderly patients for revascularisation because the risk-benefit ratio is probably more tenuous than a younger cohort. The elderly present difficulties in the evaluation of SIHD due to multiple reasons. One refinement is the adequate selection of the method to evaluate SIHD. A panoply of non-invasive techniques exists for imaging, including cardiac computed tomography, echocardiography, nuclear medicine techniques and cardiac magnetic resonance (CMR). Combining the right patient and the right method of evaluation followed by the right therapeutic strategy improves outcomes. In clinical medicine, what is important is not how much we do but how well our patients do after what we do.

CMR stress perfusion is a valuable resource with a high negative predictive value in those who do not have perfusion defects irrespective of the presence or absence of coronary artery disease.^2^ Few studies exist on the prognostic value of adenosine stress perfusion CMR in elderly patients with or without established SIHD. The study by Boonyasirinant and Kaolawanich,^3^ although not the first, is a welcome addition to the repository of existing literature that provides a cogent reason for using stress CMR for the evaluation of ischemia now addressing the elderly age group.^3^ Their findings demonstrate that while clinical data were combined with information on left ventricular function, no incremental value was seen compared to clinical data alone in predicting serious cardiac events. The presence of ischemia as detected by CMR helped predict events significantly better. The predictive power was not increased with the addition of information on late gadolinium enhancement. Esteban-Fernandez et al. found that elderly patients with a moderate or severe degree of ischemia on stress CMR have a higher risk of having an event during follow-up.^4^ Taken together, these two studies provide evidence for the use of adenosine stress CMR in predicting outcomes in elderly patients. A notable feature of both studies is the absence of significant adverse events of adenosine. However, the ISCHEMIA clinical trial reflected the limited availability of both equipment and expertise, where CMR was the least chosen method to evaluate for ischemia (5%), reflecting real-world practice.

Like almost every significant study in cardiovascular medicine, the choice of endpoints is debatable. This study defined serious cardiac events as non-fatal myocardial infarction and cardiac mortality. With current therapy, most myocardial infarctions are non-fatal, and the evolving definitions of myocardial infarction make it a moving target. Secondly, although we all try to use evidence-based medicine as the foundation of our clinical practice, we need to remember that medicine is essentially a retail business, as noted eloquently by Atul Gawande.^5^ As we treat each patient one at a time and not the population per se, statistical significance is not always synonymous with clinical relevance for the individual patient.

Another important point to remember is that the risk of adverse cardiac events, inherent in the presence of markers such as ischemia, can be mitigated by adequate OMT. No clear information on therapy used in the two groups is provided. A very significant difference in smoking rates between those with and without ischemia was noted. Although not statistically significant to be entered into the model, clinicians know how continued smoking contributes to clinical events. In real-world clinical practice, imaging is usually requested when revascularisation is contemplated in those with known SIHD and not necessarily to risk stratify patients. No technique in imaging to evaluate SIHD is the holy grail, and all are essentially complementary. With the worldwide population aging, we should seek out the best ways to evaluate and manage SIHD, and the study is a worthwhile step in this endeavour.

Dr. Thomas served as a Consultant for the ISCHEMIA study´s Clinical Coordinating Center at NYU Langone Medical Center.
